# Impact of Cold Atmospheric Plasma Pretreatment on the Recovery of Phenolic Antioxidants from Spent Coffee Grounds

**DOI:** 10.1007/s12161-024-02661-2

**Published:** 2024-08-05

**Authors:** Anastasia Kyriakoudi, Anastasia Loukri, Stamatia Christaki, Yelyzaveta Oliinychenko, Alexandros Ch. Stratakos, Ioannis Mourtzinos

**Affiliations:** 1 Laboratory of Food Chemistry and Biochemistry, Department of Food Science and Technology, Faculty of Agriculture, Forestry and Natural Environment, School of Agriculture, Aristotle University of Thessaloniki (AUTH), 54124 Thessaloniki, Greece; 2 College of Health, Science and Society, School of Applied Sciences, University of the West of England, Coldharbour Ln, Bristol, BS16 1QY UK

**Keywords:** Cold atmospheric plasma, Spent coffee grounds, Caffeine, Chlorogenic acid, Total phenolic content

## Abstract

In the present study, cold atmospheric plasma (CAP) was employed as a pretreatment method for the extraction of phenolic compounds from spent coffee grounds (SCGs). The impact of CAP treatment conditions, i.e., thickness of the SCGs layer (mm), distance between the plasma source and the SCGs layer (mm) and duration of CAP treatment (min), on the total phenol content, in vitro antioxidant activity, as well as caffeine and chlorogenic acid content of SCGs, was investigated. The process parameters were optimized with the aid of response surface methodology (RSM). After optimizing the CAP pretreatment conditions, the CAP-treated SCGs were subjected to ultrasound-assisted extraction using ethanol as the extraction solvent. The optimum conditions for CAP treatment identified, i.e., thickness, 1 mm; distance, 16 mm; and duration, 15 min, led to a significant enhancement in the recovery of bioactive compounds from SCGs compared to those obtained from untreated SCGs. Total phenolic content and antioxidant activity significantly increased (i.e., TPC from 19.0 ± 0.7 to 24.9 ± 1.4 mg GAE/100 g dry SCGs, A_DPPH_ from 106.7 ± 5.01 to 112.3 ± 4.3 μmol Trolox/100 g dry SCGs, A_ABTS_ from 106.7 ± 5.01 to 197.6 ± 5.8 μmol Trolox/100 g dry SCGs, A_CUPRAC_ from 17938 ± 157 to 18299 ± 615 μmol Trolox/100 g dry SCGs). A significant increase in caffeine content from 799.1 ± 65.1 mg to 1064 ± 25 mg/100 g dry SCGs and chlorogenic acid content from 79.7 ± 15.3 mg to 111.3 ± 3.3 mg/100 g dry SCGs, was also observed. Overall, CAP pre-treatment can be used to enhance the recovery of bioactive compounds from SCGs.

## Introduction

Coffee is the third most consumed beverage in the world, after water and tea. According to the International Coffee Organization, the global coffee consumption was estimated to be ~ 9.98 million kg during 2020–2021 (ICO 2021). The high consumption of coffee is based not only on its unique sensorial attributes but also on its beneficial health effects, e.g., decreased risk of heart diseases and certain types of cancer, and increased alertness (McNutt and He 2019). Among the ~ 100 known species of the genus *Coffea*, the species *Coffea arabica* (Arabica) and *Coffea canephora* (Robusta) are the most economically exploited ones (Esquivel and Jiménez 2012).

Considering that the coffee brewing consists of extracting certain compounds from the bean, both the industry of instant coffee as well as the domestic preparation of coffee, generate substantial quantities of by-products and wastes, mainly in the form of spent coffee grounds (SCGs), that pose significant environmental and disposal issues (Arya et al. 2022). SCGs refer to the solid material that remains after the extraction of the desirable compounds during the brewing process. They constitute a rich source of polysaccharides (i.e., cellulose, hemicellulose) (~ 50% of the dry mass of SCGs), lignin, proteins, and fats. For these reasons, till now, many coffee-producing industries utilize SCGs for purposes such as energy and biodiesel production, bioethanol production, sugar recovery, and composting (McNutt and He 2019; Campos-Vega et al. 2015). Apart from the aforementioned uses, SCGs also contain high amounts of phenolic compounds, such as caffeine and chlorogenic acid. This is why the interest of the scientific community has turned towards the recovery of these high-added value compounds employing novel approaches. The recovery of these compounds from SCGs is usually carried out with the aid of conventional solid–liquid extraction methods using organic solvents. However, these approaches are time- and energy-consuming and result in environmental threats. Moreover, conventional methods are not effective in extracting bound phenolic compounds from plant materials (Solomakou et al. 2022). For this purpose, usually, alkaline or acid hydrolysis is carried out (Titiri et al. 2023; Sarghini et al. 2021; Wongsiridetchai et al. 2018). The latter ones are not considered environmentally friendly approaches due to the use of strong alkali or acids as well as high temperatures.

In recent years, novel non-thermal technologies have emerged in the field of food science. Plasma, the fourth state of matter, is an ionized gas containing various reactive species that consist of oxygen (e.g., ozone O_3_, atomic oxygen O, excited oxygen O_2_, singlet oxygen ^1^O_2_, and superoxide anion O_2_^−^) and nitrogen (e.g., atomic nitrogen N, excited nitrogen N_2_, nitric oxide NO•) depending on the operating parameters (Mandal et al. 2018). Cold atmospheric plasma (CAP) is generated upon applying electrical or microwave energy to gases like air, helium, oxygen, hydrogen, nitrogen, argon, or mixtures of them at a temperature lower than 40 °C under atmospheric pressure (Muhammad et al. 2018). Cold plasma finds numerous applications in medicine (e.g., for wound healing, cancer treatment) as well as in food processing, e.g., for microbial inactivation due to the oxidative stress and cell membrane leakage caused by plasma reactive species. Recently, cold plasma has also been used as an innovative and emerging approach for the pre-treatment of plant materials, including agro-industrial by-products, to enhance the extraction of bioactive compounds. This is achieved through the increase in the surface roughness and surface area of the plant material as well as by regulation of the wettability of the surface (Bao et al. 2020a). Even though CAP has been employed for the enhanced recovery of phenolic compounds from some plant materials and agro-industrial by-products and wastes, e.g., grape pomace (Bao et al. 2020a), tomato pomace (Bao et al. 2020b), avocado pulp (Batista et al. 2021), green tea (Hemmati et al. 2021), and wild berries (Li et al. 2023), to the best of our knowledge, it has not been applied yet for the recovery of phenolic compounds from SCGs. The only relevant published article describes the use of CAP, generated by dielectric barrier discharge using argon as the carrier gas, as a pretreatment of SCGs towards oil recovery (Leal Vieira Cubas et al. 2020). In this view, the aim of the present study was the investigation of the impact of CAP pretreatment conditions on SCGs. For the first time, CAP generated by piezoelectric discharge technology was used to extract bioactive compounds, with air as the carrier gas. Different conditions were tested: thickness of the SCG layer (mm), distance between the plasma source and the SCG layer (mm), and duration of CAP treatment (min). These conditions were evaluated for their effects on total phenol content (TPC), in vitro antioxidant activity, and caffeine and chlorogenic acid content of SCGs. These two individual compounds were selected as they are the main phenolic compounds present in SCGs. The process parameters were optimized with the aid of response surface methodology (RSM). After optimizing the CAP pretreatment conditions, the CAP-treated SCGs were subjected to ultrasound-assisted extraction using ethanol as the extraction solvent. Scanning electron microscopy (SEM) was also employed to examine the effect of CAP treatment on the surface morphology of SCGs. Overall, the findings of the present study provide valuable insights into the potential of CAP as a pretreatment for SGCs, for their utilization as a sustainable source of bioactive compounds.

## Materials and Methods

### Spent Coffee Grounds

Spent coffee grounds from the species *Arabica* were kindly donated by a cafeteria located in Thessaloniki (Greece). The average particle size was < 0.22 mm whereas the moisture content of the SCGs was 59.3% ± 0.57 (*n* = 3). Upon their arrival at the Laboratory of Food Chemistry and Biochemistry (Thessaloniki, Greece), SCGs were stored in zip bags in the freezer (− 20 °C) till further analysis.

### Reagents and Solvents

6-Hydroxy-2,5,7,8-tetramethyl-chroman-2-acid (Trolox) (97%) and 3,4,5-trihydroxybenzoic acid (gallic acid) (99%) were purchased from Sigma-Aldrich (Stenheim, Germany). Ammonium acetate (CH_3_COONH_4_) (99%), Folin-Ciocalteu reagent, sodium carbonate (Na_2_CO_3_, 99.8%), sodium sulfate (Na_2_SO_4_) (99%), potassium chloride (KCl) (99.5%), sodium chloride (NaCl) (99.8%), sodium dihydrochloride monoacid phosphate (Na_2_HPO_4_•2H_2_O) (99.5%), and potassium dihydrogen phosphate (KH_2_PO_4_) (99.5%) were from Chem-Lab (Zedelgen, Belgium). The radical 2,2-diphenyl-1-picrylhydrazyl (DPPH^●^) (> 97%) was from TCI (Kita-Ku, Tokyo, Japan). Copper dichloride dihydrate (CuCl_2_•2H_2_O) (99.99%) was from ThermoFisher (Kandel, Germany), whereas neocuproine (2,9-dimethyl-1, 10-phenanthroline) (≥ 98%) and the bis-ammonium salt of 2,2′-azino-bis(3-ethylbenzothiazolin-6-sulfonic acid) (ABTS) (≥ 98%) were from Sigma-Aldrich GmbH (Buchs SG, Switzerland). Potassium persulfate (K_2_S_2_O_8_) was from Merck (Darmstadt, Germany). Glacial acetic acid as well as HPLC grade methanol (> 99.8%) were from Chem-Lab (Zedelgen, Belgium). Ultrahigh purity water was produced in the laboratory using a Micromatic Wasserlab system (Wasserlab, Spain).

### Experimental Design for Optimizing the CAP Treatment Conditions

**Fig. 1:**
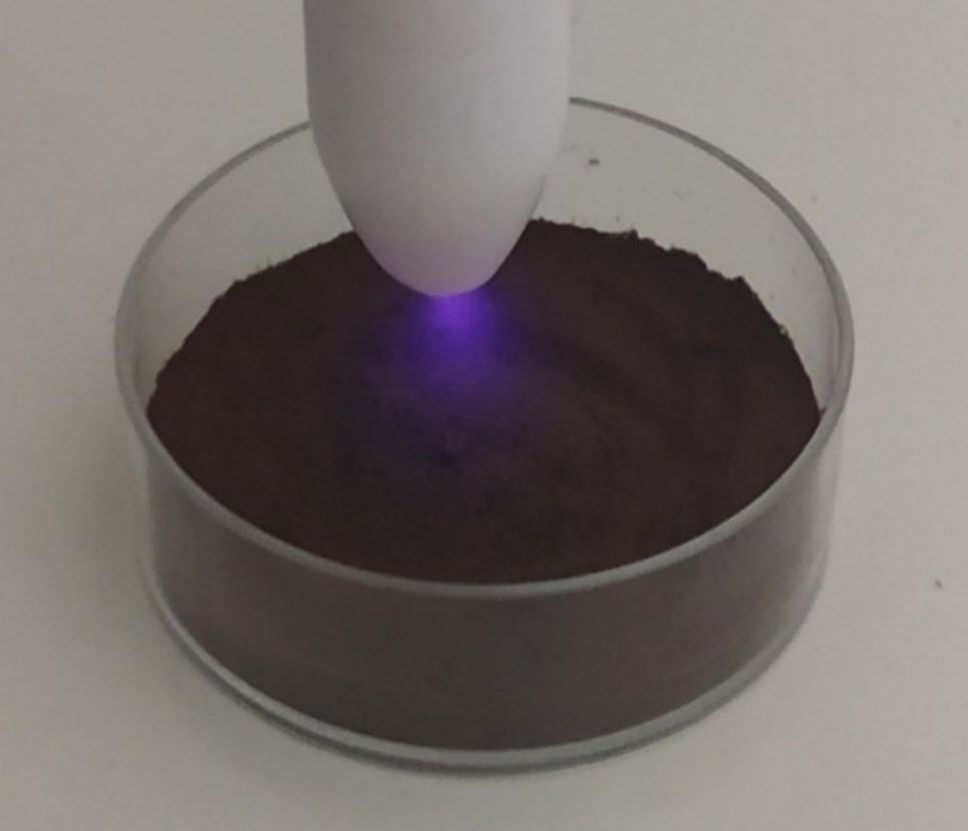
Cold atmospheric plasma treatment of SCGs

SCGs were subjected to CAP treatment under different experimental conditions. The treatment was conducted using a cold plasma, which operates on piezoelectric direct discharge technology (Piezobrush PZ2, Relyon Plasma Germany). This approach involves a direct electrical discharge from a piezo-ceramic transformer to a gas that serves as the working medium, converting a low input voltage into a high output voltage. The generator was powered by a DC voltage of 15 V, with a frequency set at 50 kHz. Atmospheric air was utilized to generate plasma, facilitated by an inbuilt axial fan for air supply. The treatments took place under at a temperature of around 23 °C and 60% relative humidity. The effect of parameters such as the thickness (mm) of the SCGs layer, the distance (mm) between the plasma source and the SCGs layer, as well as the duration (min) of the CAP treatment, were examined. Specifically, different amounts of SCGs were weighted directly into polystyrene tissue culture dishes (35 mm × 10 mm), and the surface was flattened with the aid of a spatula, in order to obtain the appropriate thickness. Then, CAP was applied at predetermined distance and duration values (Fig. 1). At the end of each pretreatment, the obtained CAP-treated SCGs were used for the recovery of phenolic compounds with the aid of ultrasound-assisted extraction using ethanol as the extraction solvent (Al-Dhabi et al. 2017). In particular, an appropriate amount of CAP-treated SCGs (~ 0.3 g) were placed in vials with screw caps and extracted with 15 mL of ethanol with the aid of an ELMASONIC S30 sonication bath operating at 37 kHz and 80W (Elma Ultrasonic Technology, Singen, Germany) for 20 min. Constant temperature (30 ± 1 °C) was maintained by adding ice periodically to the bath. At the end of the process, the mixture was centrifuged at 6000 rpm for 10 min. The supernatant was collected and stored in the freezer (− 20 °C) for further analyses.

Experiments were designed using an unblocked full factorial central composite design (CCD) of the response surface methodology with the aid of Minitab 15.1.20.0 (Minitab, Inc., State College, PA, USA) software in order to study the effects of the three independent variables of the CAP treatment, namely, thickness (mm) (*X*_1_), distance (mm) (*X*_2_), and duration (min) (*X*_3_). Each of these factors had five experimental levels. The lower and the upper levels were selected for every factor whereas the remaining ones were estimated based on the equation presented in Table 1.

**Table 1:** Levels of thickness of SCGs layer, distance between CAP source and SCGs layer, as well as duration of CAP treatment in coded and uncoded values employed in the experimental design

Symbols	Variable			Level		
		Coded value^1^				
		− a	− 1	0	+ 1	+ a
		Uncoded value^1^				
*Χ*_1_	Thickness of SCGs layer (mm)	1	2	3	4	5
*Χ*_2_	Distance between CAP source and SCGs layer (mm)	10	14	20	26	30
*Χ*_3_	Duration of CAP treatment (min)	1	5	11	17	21

^1^ $$Coded\ Value=\frac{ActualValue-\frac{(HighLevel+LowLevel)}{2}}{\frac{HighLevel-LowLevel}{2}}$$

The experimental design included 20 experimental runs, six of which were at the center points (Table 2). A wide range of responses were examined: total phenol content (TPC) (*Y*_1_), DPPH^●^ (A_DPPH_) (*Y*_2_) and ABTS^●+^ (A_ABTS_) (*Y*_3_) radical scavenging activity, cupric ion-reducing antioxidant capacity (A_CUPRAC_) (*Y*_4_), as well as the concentration of caffeine (*Y*_5_) and chlorogenic acid (*Y*_6_). Each experimental response was analyzed, and a second-order regression equation (Eq. 1) was obtained:

1 $$Y={\beta }_{0}+{\beta }_{1}{X}_{1}+{\beta }_{2}{X}_{2}+{\beta }_{3}{X}_{3+}{\beta }_{11}{X}_{12}+{\beta }_{22}{X}_{22}+{\beta }_{33}{X}_{32}+{\beta }_{12}{X}_{1}{X}_{2}+{\beta }_{13}{X}_{1}{X}_{3}+{\beta }_{23}{X}_{2}{X}_{3}$$

where *Y* corresponds to the responses, *X*_1_, *X*_2_, and *X*_3_ represent the factors of thickness, distance, and duration, and *β*_0_, *β*_1_, … *β*_23_ are the estimated coefficients with *β*_0_ being a scaling constant.

**Table 2:** Experimental design and data for 3-factor-5-level central composite design

	Independent variables			Dependent variables					
Run	Thickness of SCGs layer (mm) (*Χ*_1_)	Distance (mm) (*Χ*_2_)	Duration of CAP treatment (min) (*Χ*_3_)	TPC^a^ (mg GAE/g dry SCG)	A_DPPH_^a^ (μmol Trolox/100 g dry SCG)	A_ABTS_^a^ (μmol Trolox/100 g dry SCG)	A_CUPRAC_^a^ (μmol Trolox/100 g dry SCG)	Caffeine content^a^ (mg/100 g dry SCG)	Chlorogenic acid content^a^ (mg/100 g dry SCG)
1	2	26	5	21.0 ± 1.1	111.6 ± 14.0	221.2 ± 21.7	12628 ± 1030	782.4 ± 41.7	80.6 ± 12.8
2	1	20	11	25.0 ± 3.2	120.1 ± 0.87	217.8 ± 10.1	19647 ± 1405	848.3 ± 14.6	90.6 ± 1.7
3^b^	3	20	11	22.2 ± 2.0	118.5 ± 7.47	240.4 ± 16.4	16499 ± 1390	835.9 ± 59.9	103.0 ± 6.7
4	3	30	11	19.0 ± 0.9	110.5 ± 1.45	220.5 ± 9.8	13375 ± 553	770.8 ± 73.1	83.8 ± 4.9
5	2	14	17	23.8 ± 1.7	125.0 ± 8.31	212.5 ± 16.9	21423 ± 1889	1106.1 ± 85.4	104.2 ± 10.0
6	3	10	11	20.5 ± 0.7	115.4 ± 3.17	209.4 ± 4.68	17154 ± 709	823.4 ± 25.3	79.5 ± 1.1
7^b^	3	20	11	21.9 ± 1.7	121.9 ± 7.95	251.0 ± 11.6	17675 ± 1142	835.7 ± 50.3	100.3 ± 4.2
8	2	14	5	21.2 ± 0.4	111.7 ± 6.15	216.7 ± 15.7	12861 ± 749	843.2 ± 83.6	83.9 ± 7.3
9	3	20	1	19.4 ± 0.8	107.7 ± 5.38	204.2 ± 10.6	18672 ± 169	889.0 ± 79.4	81.5 ± 17.7
10^b^	3	20	11	24.0 ± 1.0	120.4 ± 7.42	259.5 ± 20.9	15567 ± 580	891.0 ± 45.3	107.4 ± 4.6
11	3	20	21	19.5 ± 0.2	108.6 ± 2.42	214.0 ± 16.2	16600 ± 266	823.4 ± 17.4	79.8 ± 4.6
12	4	14	5	21.3 ± 1.0	117.8 ± 4.25	211.1 ± 18.5	16519 ± 521	933.7 ± 89.5	104.3 ± 13.7
13	3	20	11	22.3 ± 0.1	119.7 ± 6.90	259.5 ± 20.7	14480 ± 1065	793.4 ± 61.3	87.3 ± 3.9
14	4	26	5	22.3 ± 1.9	121.7 ± 3.96	217.5 ± 18.1	26720 ± 1096	1129.0 ± 90.3	116.1 ± 3.3
15^b^	3	20	11	22.4 ± 2.3	118.1 ± 5.99	234.7 ± 14.9	16320 ± 919	751.1 ± 45.8	97.0 ± 2.6
16	2	26	17	21.0 ± 1.2	116.5 ± 2.48	216.4 ± 8.51	19268 ± 531	921.1 ± 1.7	101.0 ± 2.7
17	5	20	11	23.5 ± 1.7	118.4 ± 5.59	226.4 ± 18.6	18146 ± 581	899.8 ± 51.9	98.0 ± 11.0
18	4	14	17	21.4 ± 2.4	115.5 ± 14.71	207.0 ± 14.1	17743 ± 204	808.0 ± 87.1	95.0 ± 7.4
19	4	26	17	20.3 ± 0.8	108.9 ± 1.78	229.3 ± 20.4	15646 ± 716	776.6 ± 72.6	89.5 ± 12.0
20^b^	3	20	11	22.2 ± 1.2	118.8 ± 10.64	247.7 ± 19.5	16798 ± 855	763.0 ± 15.1	99.4 ± 2.6

^a^All responses are expressed as mean ± s.d. values (*n* = 3)

^b^Run nos. 3, 7, 10, 15, and 20 were the center points

The models’ significance was assessed by analysis of variance (ANOVA). The polynomial model’s fit quality was determined by the coefficient of determination (*R*^2^), the significance of each parameter through the* F* test (calculated *p* value), and the model’s lack of fit. Parameters with a *p* value less than 0.05 were deemed statistically significant. Multi-response optimization of the fitted polynomials was conducted using Minitab software. The optimum conditions identified by response surface methodology (RSM) were experimentally tested at least three times to verify if the experimental results aligned with the model’s predictions. An ultrasound-assisted extraction of untreated SCGs was also carried out for control purposes.

### Determination of the Total Phenol Content (TPC)

The total polar phenol content of the prepared CAP-treated SCGs extracts was determined spectrophotometrically by the Folin-Ciocalteu assay as previously described by Kyriakoudi et al. (2023) using a UV-1800 spectrophotometer (Shimadzu, Kyoto, Japan). Gallic acid was used as a reference standard, and results were expressed as gallic acid equivalents (mg GAE/g dry SCGs). Measurements were performed at least in triplicate, and results were expressed as the mean value ± s.d.

### In Vitro Antioxidant Activity Determination

The DPPH^●^ scavenging activity (A_DPPH_) was determined according to Kyriakoudi et al. (2023). Radical scavenging activity (%RSA) was calculated using the formula %RSA = [Abs515(*t* = 0)–Abs515(*t*)] × 100/Abs515(*t* = 0) after blank correction. ABTS^●+^ scavenging activity (A_ABTS_) was assessed following the method outlined by Re et al. (1999). Percent inhibition (% Inh) was determined using the formula % Inh = [Abs734(*t* = 0)–Abs734(*t*)] × 100/Abs734(*t* = 0) after blank correction. The cupric ion-reducing antioxidant capacity (A_CUPRAC_) of SCGs extracts was measured according to the protocol of Apak et al. (2004). Absorbance at 450 nm was recorded after 30 min. In all the above mentioned cases, measurements were performed at least in triplicate, and results were finally expressed as μmol Trolox/100 g dry SCGs with the aid of appropriate calibration curves.

### RP-HPLC–DAD Analysis of CAP-Treated SCGs Extracts

The content of caffeine and chlorogenic acid was determined by RP-HPLC–DAD. The HPLC system consisted of an Agilent 1260 Infinity II Quaternary Pump VL, an Agilent 1260 Infinity II Autosampler, and an Agilent 1260 Infinity II Diode Array Detector High Sensitivity. Separation was carried out on an InfinityLab Poroshell 120 EC-C184μm (150 × 4.6 mm i.d.) column (Agilent Technologies, Santa Clara, CA, USA). Column temperature was set at 30 ℃. The mobile phase consisted of water (0.1% CH_3_COOH) (A) and methanol (0.1% CH_3_COOH) (B). The elution protocol was 0 min; 30% (B), 0–5 min, 45% (B); 5–15 min, 65% (B); 15–18 min, 100% (B); 18–25 min, 30% (B); and 25–30 min, 30% (B). The flow rate was 0.5 mL/min and the injection volume was 10 µL. Extracts were analyzed after proper dilution (when required) and filtration through 0.45-μm PTFE filters (Frisenette, Knebel, Denmark). Monitoring was in the range 190–600 nm. Chromatographic data were processed using the OpenLab CDS version 3.5 software (2021, Agilent Technologies, Santa Clara, CA, USA). Peak identification was based on retention times and spectral characteristics (absorption maxima) with those of available standards. Quantitation of caffeine and chlorogenic acid content (mg/100 g dry SCGs) was carried out by integration of the respective peaks at 276 nm and 330 nm, respectively, with the aid of calibration curves of properly diluted methanolic solutions of available standards: (i) caffeine (*y* = 2.617*x* – 6.726, *R*^2^ = 1.000, 100–2000 ng/20 μL) and (ii) chlorogenic acid (*y* = 1.639*x* –38.032, *R*^2^ = 0.999, 50–1000 ng/20 μL).

### Surface Morphology

The surface microstructure analysis of CAP-treated under the optimized conditions as well as of untreated SCGs (control) was conducted using a FEI Quanta 650 field emission scanning electron microscope (Hillsboro, Oregon, USA). Samples were prepared for SEM imaging by first mounting them onto aluminum stubs with adhesive carbon tape. To improve conductivity and reduce charging effects during imaging, a thin layer of gold was then sputter-coated over the samples. The SEM images were acquired by applying an acceleration voltage of 1–2 kV at low vacuum conditions using 350 × , 10,000 × , and 20,000 × magnification.

### Microbiogical Analysis

An appropriate amount (10 g) of CAP-treated SCGs under the optimum experimental conditions, were transferred into sterile stomacher plastic bags with 90 mL of minimum required diluent (MRD) and blended with a stomacher (400 Circulator Lab Blender) for 1 min at 240 rpm. The samples were prepared in a series of decimal dilutions with sterile MRD solution, and 100 µm of each dilution was spread on the plates. Total microbial counts (TVC), yeasts, and molds were evaluated using suitable growth media and incubation conditions. TVC were enumerated using plate count agar (Oxoid, CM0325B) at 32 °C for a 48-h incubation. Yeasts and molds were determined using potato dextrose agar (Oxoid, CM0139) at 25 °C for a 120-h incubation. Microbial counts were expressed as log colony forming units (CFU) per gram (CFU/g).

### Statistical Analysis

Data obtained by the experimental runs were analyzed by one-way analysis of variance (ANOVA) using the Minitab 15.1.20.0 (Minitab, Inc., State College, PA, USA) software. Moreover, a two-tailed paired *t* test (*p* = 0.05) was conducted using the IBM SPSS, Version 27.0 (IBM Corp., Armonk, NY, USA) in order to identify significant differences between the mean values of TPC, A_DPPH_, A_ABTS_, A_CUPRAC_, and caffeine and chlorogenic acid content of the extracts obtained without any CAP pretreatment (control) and with the CAP pretreatment under the optimum conditions.

## Results and Discussion

### Model Fitting for TPC, ADPPH, AABTS, ACUPRAC, and Caffeine and Chlorogenic Acid Content

Experimental responses for all the examined variables are presented in Table 2. As can be observed, the TPC content ranged from 19.4 to 25.0 mg GAE/g dry SCGS, the A_DPPH_ values ranged from 107.7 to 125.0 μmol Trolox/100 g dry SCG, the A_ABTS_ values varied from 204.2 to 259.5 μmol Trolox/100 g dry SCG, the A_CUPRAC_ values from 12,861 to 26,720 μmol Trolox/100 g dry SCG, the caffeine content values varied from 770.8 to 1129.0 mg/100 g dry SCG, and the chlorogenic acid values were found to range from 79.5 to 170.4 mg/100 g dry SCG.

The experimental responses (Table 2) were analyzed by ANOVA (Table 3) to test the validity of each model. According to Table 3, the models *Y*_1_–*Y*_6_ for TPC, A_DPPH_, A_ABTS_, A_CUPRAC_, and caffeine and chlorogenic acid content, respectively, showed a statistically significant regression, whereas the lack-of-fit was not significant indicating the models’ suitability in determining the optimal conditions. The *R*^2^ values ranged from 0.882 to 0.910, indicating a high correlation between the measured and the predicted values since according to literature, for a satisfactory fit of the model, *R*^2^ must be greater than 0.75 (Le Man et al. 2010). The polynomial equations (Eqs. 2–7) representing the relationship between each response and the examined variables (models Y_1_–Y_6_) are shown in Table 4.

**Table 3:** Results of ANOVA for the TPC, A_DPPH_, A_ABTS_, A_CUPRAC_, and caffeine and chlorogenic acid content values

	TPC	A_DPPH_	A_ABTS_	A_CUPRAC_	Caffeine content	Chlorogenic acid content
*R*^2^ (%)	90.56	90.64	89.42	88.23	91.64	91.43
*R*^2^_adj_ (%)	82.06	82.21	79.91	77.64	84.12	83.71
	*p* values					
Regression	0.000	0.000	0.001	0.001	0.000	0.000
Lack of fit	0.644	0.098	0.953	0.290	0.397	0.114
Linear coefficients						
*Χ*_1_	0.016	0.380	0.071	0.175	0.011	0.500
*Χ*_2_	0.006	0.026	0.002	0.894	0.087	0.018
*Χ*_3_	0.000	0.000	0.020	0.007	0.001	0.000
Quadratic coefficients						
*Χ*_1_^2^	0.003	0.557	0.001	0.023	0.008	0.052
*Χ*_2_^2^	0.001	0.013	0.000	0.159	0.363	0.001
*Χ*_3_^2^	0.000	0.000	0.000	0.017	0.109	0.346
Interactive coefficients						
*Χ*_1_*Χ*_2_	0.151	0.343	0.379	0.474	0.004	0.034
*Χ*_1_*Χ*_3_	0.039	0.000	0.463	0.015	0.000	0.000
*Χ*_2_*Χ*_3_	0.030	0.009	0.500	0.488	0.003	0.072

**Table 4:** Polynomial equations for TPC, A_DPPH_, A_ABTS,_ A_CUPRAC_, and caffeine and chlorogenic acid content responses

Model	Response	Uncoded value of factors
*Y*_1_	TPC	TPC = 13.52 − 3.51*X*_1_ + 0.87*X*_2_ + 1.21*X*_3_ + 0.51*X*_1_^1^ − 0.02*X*_2_^2^ − 0.03*X*_3_2 − 0.09*X*_1_*X*_3_ − 0.02*X*_2_*X*_3_
*Y*_2_	A_DPPH_	A_DPPH_ = 66.23 + 2.00*X*_2_ + 5.56*X*_3_ − 0.04*X*_2_^2^ − 0.09*X*_3_^2^ − 0.69*X*_1_*X*_2_ − 0.07*X*_2_*X*_3_
*Y*_3_	A_ABTS_	A_ABTS_ = 38.49 + 12.18*X*_2_ + 6.71X_3_ − 6.59*X*_1_^2^ − 0.33X_2_^2^ − 0.39*X*_3_^2^
*Y*_4_	A_CUPRAC_	A_CUPRAC_ = 16,298.6 + 1120.0*X*_3_ + 557.80*X*_1_^2^ − 22.60*X*_3_^2^ − 184.0*X*_1_*X*_3_
*Y*_5_	Caffeine content	Caffeine content = 623.69 − 152.70*X*_1_ + 42.65*X*_3_ + 17.42*X*_1_^2^ + 5.89*X*_1_*X*_2_ − 8.27*X*_1_*X*_3_ − 1.05*X*_2_*X*_3_
*Y*_6_	Chlorogenic acid content	Chlorogenic acid content = 0.67 + 2.53*X*_2_ + 4.83*X*_3_ − 0.07*X*_2_^2^ + 0.35*X*_1_*X*_2_ − 1.01*X*_1_*X*_3_

### Main Effects of CAP Pretreatment Conditions on Total Phenol Content, Antioxidant Activity, and Caffeine and Chlorogenic Acid Content

#### The Effect of Thickness of SCGs Layer

**Fig. 2:**
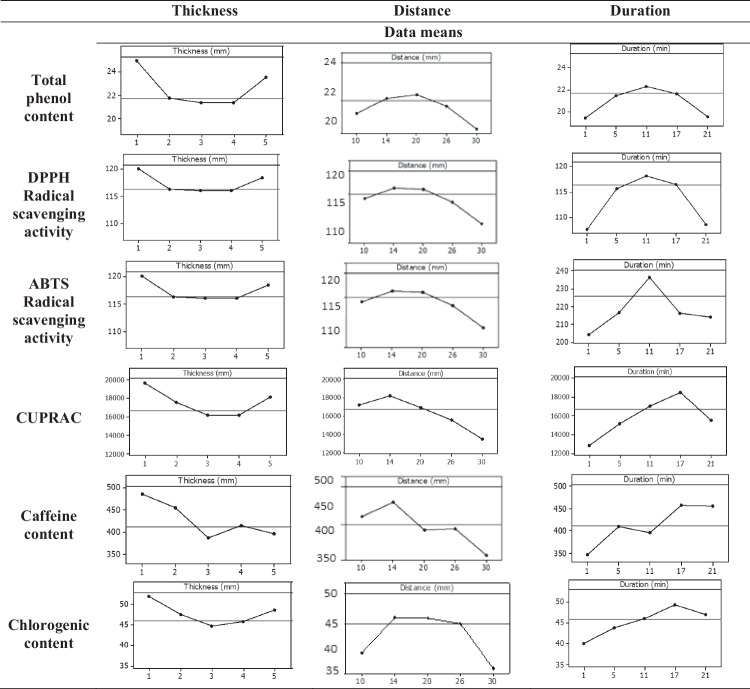
Main effects plots illustrating the effect of thickness (mm) of the SCGs layer, the distance (mm) between the plasma source and the SCGs layer and the duration (min) of CAP treatment on total phenol content, DPPH^●^ scavenging activity, ABTS^●+^ scavenging activity, cupric ion-reducing antioxidant capacity, as well as caffeine and chlorogenic acid content

In the present study, the effect of thickness of the SCGs layer during CAP treatment on the TPC, A_DPPH_, A_ABTS_, A_CUPRAC_, and caffeine and chlorogenic acid content values was investigated. The thickness of the SCGs layer, as illustrated in the main effects plots in Fig. 2, varied from 1 to 5 mm. The results indicated that the optimum responses for TPC, A_DPPH_, A_ABTS_, A_CUPRAC_, and caffeine and chlorogenic acid content were achieved at a thickness of 1 mm. Increasing the thickness beyond this point resulted in a decrease in all examined responses. To the best of our knowledge, there is extremely limited information regarding the effect of thickness of plant materials during CAP treatment on the extraction efficiency of bioactive compounds. However, Zhao et al. (2023), who applied cold plasma to enhance the extraction yield of the diterpenes taxanes from *Taxus cuspidata*, correlated sample loading weight with thickness and concluded that when the loading weight was lower, degradation of taxanes could occur to some extent since a small amount of sample was intensively treated with cold plasma.

#### The Effect of Distance Between Plasma Source and SCGs Layer

Generally, the distance between the plasma source and the sample layer can have a significant effect on the recovery of phenolic compounds from plant materials during CAP treatment, considering that when the plasma source is close to the treated area, the arcing phenomenon is generated. During arching, the local temperature increases, and the cold plasma has a negative impact on the treated sample (Kumar et al. 2023; Nemchinsky and Severance 2006). Moreover, this distance determines the exposure level of the sample surface to reactive species generated by the plasma, which in turn affects the properties of the matrix. The mechanism of action of CAP involves the generation of reactive oxygen and nitrogen species (RONS), which can lead to cell membrane/wall disruption, oxidation of cellular components, etc. The intensity of these effects is strongly influenced by the proximity of the matrix to the plasma source. In this view, in the present study, the impact of distance between plasma source and SCGs layer on the TPC, A_DPPH_, A_ABTS_, A_CUPRAC_, and caffeine and chlorogenic acid content values is visualized in Fig. 2 as main effects plots. In particular, the distance between plasma source and SCGs layer ranged from 1 to 30 mm. As can be seen in the respective plots in Fig. 2, increase in the distance up to 20 mm results in an increase in TPC, antioxidant activity, as well as caffeine and chlorogenic acid content. However, further increase up to the highest examined level (i.e., 30 mm) resulted in a decrease in all the examined responses. These findings cannot be easily compared with those in literature because, to the best of our knowledge, in the majority of the published articles dealing with the CAP pretreatment for the extraction of bioactive compounds from plant materials, the distance between the plasma source and the material is usually kept constant (e.g., Leal Vieira Cubas et al. 2020). It is worth mentioning that the impact of CAP on food quality attributes, such as color, texture, and nutritional content, varies with the distance from the plasma source. The density of reactive species increases closer to the plasma source and thus excessive exposure to ROS and NOS due to very short distances could lead to undesirable changes, such as nutrient degradation (Zhang et al. 2019).

#### The Effect of Duration of CAP Treatment

Analysis of variance for TPC, A_DPPH_, A_ABTS_, A_CUPRAC_, and caffeine and chlorogenic acid content values (Table 3) showed that duration of CAP treatment had a significant effect on all the examined variables. In particular, the effect of duration of CAP treatment on the examined responses is visualized in the form of main effects plots (Fig. 2). In the present study, duration ranged from 1 to 21 min. As can be seen, an increase in the duration of up to 17 min results in an increase in TPC, antioxidant activity, as well as caffeine and chlorogenic acid content. However, further increase up to the highest examined level (i.e., 21 min) resulted in a decrease in all the examined responses. Similar observations have also been reported by Hemmati et al. (2021), who investigated the impact of CAP on total phenolic and flavonoid content, antioxidant activity, and surface morphology of green tea powder. These authors examined different exposure times (i.e., 2, 4, 6, and 8 min) and concluded that total phenol and flavonoid content as well as DPPH radical scavenging activity decreased drastically by increasing exposure time. Reduced total phenol content values upon prolonged CAP treatment were also observed for orange juice (Almeida et al. 2015). These authors attributed this finding to the fact that energetic electrons generated by plasma discharge decompose oxygen molecules into single oxygen atoms which then react with oxygen molecules to form ozone. The latter one is believed to degrade phenolic compounds by rupturing their aromatic rings. The same trend has also been reported for the TPC of apple juice (Liao et al. 2018), white grape juice (Pankaj et al. 2017), as well as tomato juice (Ali et al. 2021). However, some studies have reported an increase in phenolic content and antioxidant activity upon CAP treatment. More specifically, Tappi et al. (2018) found an increase in the antioxidant activity of fresh-cut apples after 10-min CAP treatment. The authors attributed this increase to the catechin oxidation that resulted in the formation of procyanidins, which exhibit higher antioxidant activity than the initial compounds.

#### The Effect of Interactions Among Thickness, Distance, and Duration

**Fig. 3:**
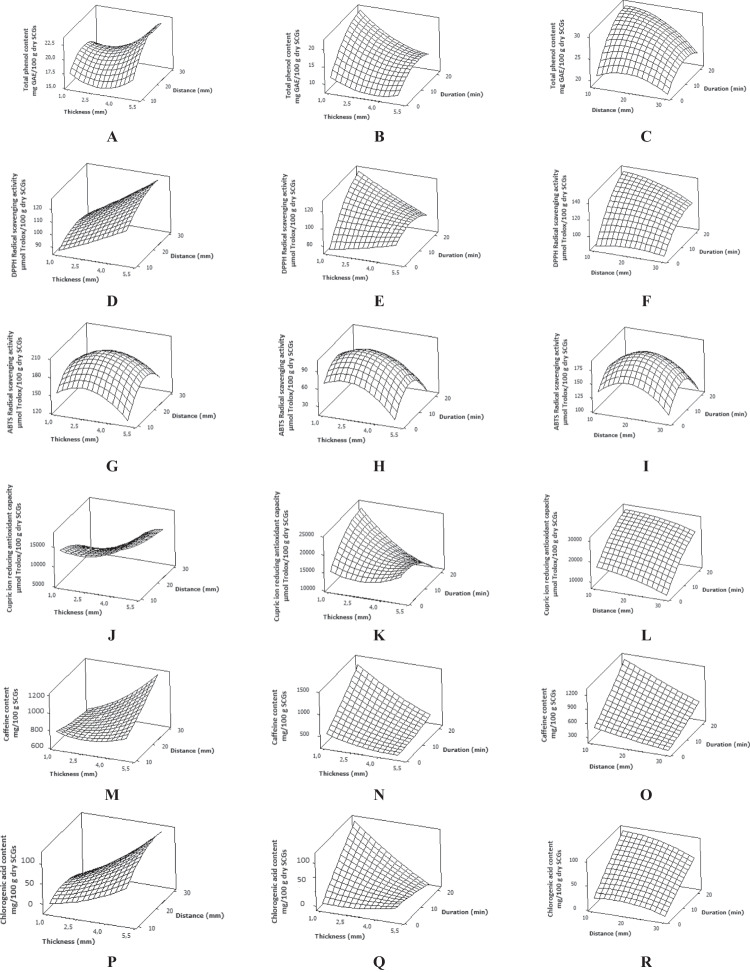
Surface plots for total phenol content (**A**, **B**, **C**), DPPH^●^ scavenging activity (**D**, **E**, **F**), ABTS^●+^ scavenging activity (**G**, **H**, **I**), cupric ion-reducing antioxidant capacity (**J**, **K**, **L**), as well as caffeine (**M**, **N**, **O**) and chlorogenic acid content (**P**, **Q**, **R**) values affected by thickness, distance, and duration of CAP pretreatment. In all instances, the third factor was maintained at its middle level

RSM is a powerful statistical tool to investigate the interactions among all the examined variables. In particular, the effects of two paired factors on the responses can be visualized by three-dimensional response surface plots that derive from the above mentioned model equations. Figure 3A–R illustrates the effects of the examined independent variables on the TPC, antioxidant activity, as well as on caffeine and chlorogenic acid content. As can be observed, most of the responses exhibited a similar trend in the response surface plots. More specifically, according to the results in Table 3, the interaction between the thickness and duration (i.e., *X*_1_*X*_3_ term) was found to be significant for all the responses apart from the A_ABTS_. In this view, the shape of the respective response surface plots (Fig. 3) indicates that the highest values of TPC and major individual phenolic compounds as well as antioxidant activity were achieved by keeping thickness at its low level (i.e., ~ 1 mm) while increasing the duration of CAP treatment up to around its middle level (i.e., ~ 15 min). It can also be observed that after this rise, all the examined responses tend to decline for reasons described in detail above. Regarding the term *X*_2_*X*_3_ which describes the interactions between distance and duration of CAP treatment, it was found to be significant for TPC, A_DPPH_, and caffeine content. As can be observed from the respective surface plots (Fig. 3), TPC, antioxidant activity, as well as caffeine and chlorogenic acid content increase by adopting a short distance between the plasma source and the SCGs (~ 16 mm) while keeping the duration of CAP treatment around its middle level (~ 15 min).

#### Multiple Response Optimization for CAP Treatment Conditions

A multiple response optimization approach for the independent variables (i.e., thickness, distance, duration) based on TPC, A_DPPH_, A_ABTS_, A_CUPRAC_, and caffeine and chlorogenic acid content values were applied. The optimum combinations of the thickness, distance, and duration is presented in Table 5. As can be observed, the predicted values for the above mentioned variables were found to fit well with the experimental ones of the respective responses, confirming the validity and adequacy of the predicted models. In additions, for comparison purposes, an ultrasound-assisted extraction of untreated SCGs was also carried out. For untreated samples, the values of TPC, A_DPPH_, A_ABTS_, A_CUPRAC_, and caffeine and chlorogenic acid content of the obtained extract, i.e., 19.0 ± 0.7 mg GAE/100 g dry SCGs, 106.7 ± 5.01 μmol Trolox/100 g dry SCGs, 199.1 ± 9.7 μmol Trolox/100 g dry SCGs, 17,938 ± 157 μmol Trolox/100 g dry SCGs, 799.1 ± 65.1 mg/100 g dry SCGsm, and 79.7 ± 15.3 mg/100 g dry SCGs, respectively, were found to be significantly lower (*p* < 0.05) according to* t* test [TPC: *t*_experimental_ (3.27) > *t*_critical_ (2.31), A_DPPH_: *t*_*e*xperimental_ (6.36) > *t*_critical_ (2.31), A_ABTS_: *t*_experimental_ (125.89) > *t*_critical_ (2.31), A_CUPRAC_: *t*_experimental_ (149.96) > *t*_critical_ (2.31), caffeine content: *t*_experimental_ (14.01) > *t*_critical_ (2.31), and chlorogenic acid content: *t*_experimental_ (27.84) > *t*_critical_ (2.31)] compared to those shown in Table 5, after the CAP-treatment at the optimum conditions. As it can be observed, the CAP pretreatment resulted in the enhancement of the recovery of the bioactive compounds of SCGs.

**Table 5:** Optimum values of the thickness, distance, and duration, as well as predicted and experimental response values

Factor	Optimum actual values	Predicted values	Mean experimental values^a^
Thickness of SCGs layer (mm)	1	TPC (mg GAE/100 g dry SCGs)	
		26.1	24.9 ± 1.4
		A_DPPH_ (μmol Trolox/100 g dry SCGs)	
		127.3	112.3 ± 4.3
Distance between CAP source and SCGs layer (mm)	16	A_ABTS_ (μmol Trolox/100 g dry SCGs)	
		208.3	197.6 ± 5.8
		A_CUPRAC_ (μmol Trolox/100 g dry SCGs)	
		23,164	18,299 ± 615
Duration of CAP treatment (min)	15	Caffeine content (mg/100 g dry SCGs)	
		1153	1064 ± 25
		Chlorogenic acid content (mg/100 g dry SCGs)	
		113.6	111.3 ± 3.3

^a^All responses are expressed as mean ± s.d. values (*n* = 3)

#### Effect of CAP Treatment on Surface Microstructure

**Fig. 4:**
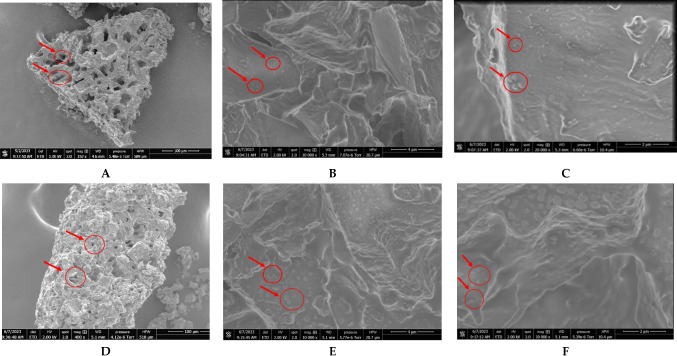
Micrographs of untreated SCGs (control) (**A**–**C**) and CAP-treated SCGs upon the optimum conditions as described above (**D**–**F**), obtained by SEM. Magnification, 400 (**A**), 10,000 (**B**), and 20,000 (**C**)

SEM was employed in order to visualize the effect of CAP treatment on the surface morphology of SCGs as shown in Fig. 4. In particular, SCGs treated with CAP under the optimum conditions (i.e., thickness, 1 mm; distance, 16 mm; and duration, 15 min), as well as untreated (control) ones, were examined and exhibited evident porosity and granular morphology at a magnification of 400 × , when the pores seemed degraded (Fig. 4A and D). At higher magnification, it was evidenced that there was a noticeable increase in evaporated aqueous droplets on the surface of the SCGs treated with CAP for 15 min (see Fig. 4E and F) compared to the untreated ones (Fig. 4B and C). Considering that the SCGs and their surrounding tissue have a barrier function that prevents moisture evaporation, excessive evaporation caused after CAP treatment can be considered as an indication of the fracture of the cell wall integrity. In the same context, Bao et al. (2020a) carried out a 15-min high voltage CAP treatment on tomato pomace towards improving phenolics extractability. The authors concluded that after the CAP treatment, the samples exhibited less or no visible cell wall structures and lost their cell integrity, as revealed by the obtained SEM images. The same research group applied a 15-min high voltage CAP treatment also on grape pomace, and they revealed the presence of rupture fragments after SEM analysis (Bao et al. 2020b). Such fragments and cracks have also been reported in the case of SCGs for oil extraction (Leal Vieira Cubas et al. 2020). These authors attributed these findings to the fact that the reactive species (e.g., peroxides, superoxides, anions) generated from plasma, may break down hydrogen or other non-covalent bonds of cell wall polymers. Moreover, as can be seen in Fig. 4C and F, the untreated SCGs showed smoother and flatter surfaces, while the CAP-treated SCGs exhibited an increased surface roughness. Similar observations have also been made by Li et al. (2023) who examined the effect of CAP pretreatment for the enhancement of anthocyanins from haskap, a wild berry.

Considering all the above, it is evident that after CAP treatment, the penetration of extraction solvents can be significantly higher resulting in the enhancement of mass transfer with a concomitant decrease in extraction time as well as the quantity of solvents used. In this view, CAP constitutes a promising pretreatment approach towards the “green” extraction of valuable phenolic compounds from SCGs.

#### Microbial Load

Table 6 shows the effect of CAP treatment on microbial populations of SCGs. Total viable counts were significantly reduced by CAP treatment. Following a 5 min of treatment, no TVC was detected, indicating a decontamination effect attributable to CAP treatment. These findings align with existing literature, where CAP treatment has shown consistent antimicrobial effects across various food powders. For instance, studies have reported significant reductions in bacterial populations in green tea powder (Hemmati et al. 2021), herbal tea powder (Chingsungnoen et al. 2018), onion powder (Kim et al. 2017), black pepper powder (Mošovská et al. 2018), and oregano powder (Hertwig et al. 2015). Furthermore, our research revealed no detectable levels of yeasts and molds in both CAP-treated and untreated SCGs (Table 1). The antimicrobial efficacy of CAP is primarily attributed to the wide range of reactive species produced through the application of cold plasma treatment. According to Varilla et al. (2020), the reactive species include charged particles, radicals, and UV photons. These reactive species collectively play a role in the degradation of microbial cells through diverse mechanisms, such as oxidative stress, membrane impairment, and disruption of DNA/RNA (Lv and Cheng 2022). Collectively, these results highlight the efficacy of CAP treatment in achieving microbial decontamination across various food powders, including SCGs, and underscore its potential as a decontamination method.

**Table 6:** TVC and yeasts and molds in untreated and CAP-treated SCGs for 5, 10, and 15 min

Microorganisms	0 min	5 min	10 min	15 min
TVC levels (log CFU/g)	2.60 ± 0.1	n.d	n.d	n.d
Yeasts and mold levels (log CFU/g)	n.d.^*^	n.d.^*^	n.d.^*^	n.d.^*^

^*^n.*d*., Below the detection limit (2 log CFU/g)

## Conclusions

This study is the first to use piezoelectric discharge technology to produce cold atmospheric plasma for pretreating spent coffee grounds (SCGs) to extract phenolic compounds. The impact of CAP treatment conditions, i.e., the thickness of the SCGs layer, the distance between the plasma source and the SCGs layer and duration of CAP treatment, on the total phenol content, in vitro antioxidant activity, as well as caffeine and chlorogenic acid content of SCGs, was investigated using RSM. Thickness, 1 mm; distance, 16 mm; and duration, 15 min, were found to be the optimum CAP treatment conditions. The outcomes of the present study underscore the need for careful optimization of CAP processes in order to ensure the maximization of bioactive compound recovery while minimizing potential adverse effects on bioactive properties. Overall, the proposed CAP pretreatment approach could be of use towards the valorization of this coffee brewing by-product as a sustainable source of valuable compounds. Future research should focus on developing standardized guidelines for CAP treatment conditions tailored to specific food by-products and wastes.

## Data Availability

No datasets were generated or analysed during the current study.
